# 3-Chloro-4-(4-chloro­phen­oxy)-*N*-[(*Z*)-(5-nitro­thio­phen-2-yl)methyl­idene]aniline

**DOI:** 10.1107/S1600536812001316

**Published:** 2012-01-14

**Authors:** Gonca Özdemir Tarı, Şamil Işık

**Affiliations:** a Department of Physics, Faculty of Arts and Sciences, Ondokuz Mayıs University, Kurupelit, TR-55139 Samsun, Turkey

## Abstract

In the title compound, C_17_H_10_Cl_2_N_2_O_3_S, the thio­phene ring and the central benzene ring are almost coplanar [dihedral angle = 8.44 (3)°], while the dihedral angle between the two benzene rings rings is 77.49 (9)°. The crystal packing is stabilized by inter­molecular C—H⋯O hydrogen bonds.

## Related literature

For background to the properties and uses of Schiff bases, see: Barton & Ollis (1979 ▶); Layer (1963 ▶); Ingold (1969 ▶); Cohen *et al.* (1964 ▶). For comparative bond lengths, see: Özdemir Tarı *et al.* (2011 ▶); Kazak *et al.* (2000 ▶); Aygün *et al.* (1998 ▶).
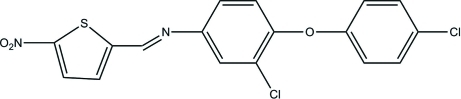


## Experimental

### 

#### Crystal data

C_17_H_10_Cl_2_N_2_O_3_S
*M* *_r_* = 393.23
Monoclinic, 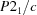

*a* = 16.3698 (9) Å
*b* = 6.7787 (2) Å
*c* = 15.9609 (9) Å
β = 105.284 (4)°
*V* = 1708.47 (14) Å^3^
*Z* = 4
Mo *K*α radiation
μ = 0.52 mm^−1^
*T* = 293 K
0.45 × 0.30 × 0.05 mm

#### Data collection

Stoe IPDS II diffractometer
Absorption correction: integration (*X-RED32*; Stoe, 2002) ▶ *T*_min_ = 0.817, *T*_max_ = 0.942
10905 measured reflections
3343 independent reflections
2317 reflections with *I* > 2σ(*I*)
*R*_int_ = 0.062

#### Refinement

*R*[*F* ^2^ > 2σ(*F* ^2^)] = 0.050
*wR*(*F* ^2^) = 0.124
*S* = 1.01
3343 reflections
226 parameters
H-atom parameters constrained
Δρ_max_ = 0.73 e Å^−3^
Δρ_min_ = −0.26 e Å^−3^

### 

Data collection: *X-AREA* (Stoe, 2002) ▶; cell refinement: *X-AREA* ▶; data reduction: *X-RED32* (Stoe, 2002) ▶; program(s) used to solve structure: *SHELXS97* (Sheldrick, 2008 ▶); program(s) used to refine structure: *SHELXL97* (Sheldrick, 2008 ▶); molecular graphics: *ORTEPIII* (Burnett & Johnson, 1996 ▶), *ORTEP-3 for Windows* (Farrugia, 1997 ▶) and *PLATON* (Spek, 2009 ▶); software used to prepare material for publication: *SHELXL97*.

## Supplementary Material

Crystal structure: contains datablock(s) I, global. DOI: 10.1107/S1600536812001316/bt5785sup1.cif

Structure factors: contains datablock(s) I. DOI: 10.1107/S1600536812001316/bt5785Isup2.hkl

Supplementary material file. DOI: 10.1107/S1600536812001316/bt5785Isup3.cml

Additional supplementary materials:  crystallographic information; 3D view; checkCIF report

## Figures and Tables

**Table 1:** Hydrogen-bond geometry (Å, °)

*D*—H⋯*A*	*D*—H	H⋯*A*	*D*⋯*A*	*D*—H⋯*A*
C3—H3⋯O2^i^	0.93	2.38	3.278 (3)	162
C13—H13⋯O1^ii^	0.93	2.53	3.369 (4)	150

Symmetry codes: (i) 
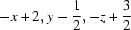
; (ii) 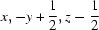.

**Fig. 1.**
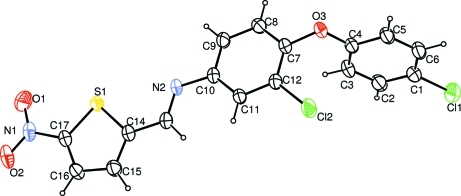
The molecular structure of the title compound, showing the atom-numbering scheme and 30% probability diplacement ellipsoids.

**Fig. 2.**
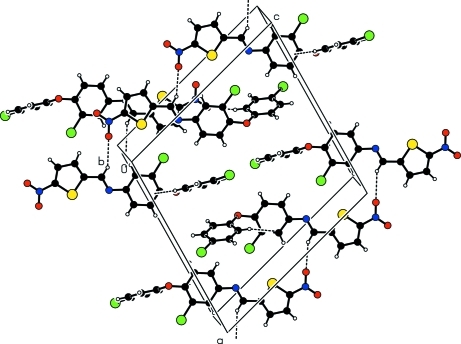
The crystal packing of the title compound. Intermolecular hydrogen bonds are shown as dashed lines.

## supplementary crystallographic information

### Comment

Schiff bases are used as starting materials in the synthesis of important drugs, such as antibiotics and antiallergic, antiphlogistic, and antitumor substances (Barton *et al.*, 1979; Layer, 1963; Ingold 1969). They show photochromism and thermochromism (Cohen *et al.*, 1964).

In this paper, the structure of the title compound, is reported. The N2=C13 bond length of 1.269 (3)Å is typical of a double bond, which is similar to the corresponding bond length in 2-[(4-Hydroxyphenyl)iminomethyl]-thiophene [1.282 (2) Å; Kazak *et al.*, 2000]; 3-methoxy-2-[(*E*)-(4-methoxyphenyl)imino)methyl)phenol [1.278 (3) Å, Özdemir Tarı *et al.*, 2011] and *N*-(2,4-dinitrophenyl)-*N*-methylhydrozone [1.279 (2) Å, Aygün *et al.*, 1998].

The N2—C10 bond distance is 1.419 (3) Å, which is in good agreement with the corresponding bond lengths in 2-[(4-Hydroxyphenyl)iminomethyl]-thiophene [1.422 (2) Å, Kazak *et al.*, 2000] and 3-methoxy-2-[(*E*)-(4-methoxyphenyl) imino)methyl)phenol [1.419 (2) Å, Özdemir Tarı *et al.*, 2011].

The C14—S1 and the C17—S1 distance are 1.713 (3)Å and 1.707 (2) Å, respectively. These distances are in good agreement with a related compound [1.712 (2)Å and 1.705 (3) Å; Kazak *et al.*, 2000]. The S···N2 distance is 3.036Å agree with similar length in related compound [3.135 (2) Å; Kazak *et al.*,2000].

Thermochromism or photochromism depends on the planarity or non-planarity of the molecule, respectively. The title compound might have photochromic properties because of the non-planarity of the molecule. The crystal structure is stabilized by intermolecular C—H···O hydrogen bonds.

### Experimental

The compound (*Z*)—*N*-[3-chloro-4-(4-chlorophenoxy)phenyl]-1-(5-nitrothiophen-2-yl) methanimine was prepared by refluxing a mixture of a solution containing 5-nitro-2-thiophene-carboxaldehyde (0.077 g 0.049 mmol) in 20 ml ethanol and a solution containing 3-Chloro-4-(4-Chloro-phenoxy)aniline (0.012 g 0.049 mmol) in 20 ml ethanol. The reaction mixture was stirred for 1 h under reflux. The crystals of (*Z*)—*N*-[3-chloro-4-(4-chlorophenoxy)phenyl]-1-(5-nitrothiophen-2-yl)methanimine suitable for X-ray analysis were obtained from ethylalcohol by slow evaporation (yield % 78; m.p 402–404K).

### Crystal data

**Table:**

C_17_H_10_Cl_2_N_2_O_3_S	*F*(000) = 800
*M**_r_* = 393.23	*D*_x_ = 1.529 Mg m^−^^3^
Monoclinic, *P*2_1_/*c*	Mo *K*α radiation, λ = 0.71073 Å
Hall symbol: -P 2ybc	Cell parameters from 14032 reflections
*a* = 16.3698 (9) Å	θ = 1.3–28.4°
*b* = 6.7787 (2) Å	µ = 0.52 mm^−^^1^
*c* = 15.9609 (9) Å	*T* = 293 K
β = 105.284 (4)°	PRISM, brown
*V* = 1708.47 (14) Å^3^	0.45 × 0.30 × 0.05 mm
*Z* = 4	

### Data collection

**Table:**

Stoe IPDS II diffractometer	3343 independent reflections
Radiation source: fine-focus sealed tube	2317 reflections with *I* > 2σ(*I*)
graphite	*R*_int_ = 0.062
Detector resolution: 6.67 pixels mm^-1^	θ_max_ = 26.0°, θ_min_ = 2.6°
φ scan rotation method	*h* = −20→20
Absorption correction: integration (*X-RED32*; Stoe, 2002)	*k* = −7→8
*T*_min_ = 0.817, *T*_max_ = 0.942	*l* = −19→16
10905 measured reflections	

### Refinement

**Table:**

Refinement on *F*^2^	Primary atom site location: structure-invariant direct methods
Least-squares matrix: full	Secondary atom site location: difference Fourier map
*R*[*F*^2^ > 2σ(*F*^2^)] = 0.050	Hydrogen site location: inferred from neighbouring sites
*wR*(*F*^2^) = 0.124	H-atom parameters constrained
*S* = 1.01	*w* = 1/[σ^2^(*F*_o_^2^) + (0.0716*P*)^2^] where *P* = (*F*_o_^2^ + 2*F*_c_^2^)/3
3343 reflections	(Δ/σ)_max_ = 0.001
226 parameters	Δρ_max_ = 0.73 e Å^−^^3^
0 restraints	Δρ_min_ = −0.26 e Å^−^^3^

### Special details

**Table:**

Geometry. All e.s.d.'s (except the e.s.d. in the dihedral angle between two l.s. planes) are estimated using the full covariance matrix. The cell e.s.d.'s are taken into account individually in the estimation of e.s.d.'s in distances, angles and torsion angles; correlations between e.s.d.'s in cell parameters are only used when they are defined by crystal symmetry. An approximate (isotropic) treatment of cell e.s.d.'s is used for estimating e.s.d.'s involving l.s. planes.
Refinement. Refinement of *F*^2^ against ALL reflections. The weighted *R*-factor *wR* and goodness of fit *S* are based on *F*^2^, conventional *R*-factors *R* are based on *F*, with *F* set to zero for negative *F*^2^. The threshold expression of *F*^2^ > σ(*F*^2^) is used only for calculating *R*-factors(gt) *etc*. and is not relevant to the choice of reflections for refinement. *R*-factors based on *F*^2^ are statistically about twice as large as those based on *F*, and *R*- factors based on ALL data will be even larger.

### Fractional atomic coordinates and isotropic or equivalent isotropic displacement parameters (Å^2^)

**Table:**

	*x*	*y*	*z*	*U*_iso_*/*U*_eq_	
C1	0.58285 (16)	−0.1486 (5)	0.16411 (18)	0.0613 (8)	
C2	0.63253 (18)	−0.2004 (5)	0.2450 (2)	0.0652 (8)	
H2	0.6545	−0.3273	0.2557	0.078*	
C3	0.64914 (17)	−0.0589 (5)	0.31044 (18)	0.0605 (8)	
H3	0.6824	−0.0910	0.3655	0.073*	
C4	0.61642 (14)	0.1292 (4)	0.29387 (16)	0.0498 (6)	
C5	0.56516 (16)	0.1787 (5)	0.21356 (17)	0.0566 (7)	
H5	0.5422	0.3047	0.2032	0.068*	
C6	0.54833 (17)	0.0374 (5)	0.14815 (18)	0.0622 (8)	
H6	0.5137	0.0684	0.0935	0.075*	
C7	0.69762 (15)	0.2549 (4)	0.42938 (16)	0.0468 (6)	
C8	0.68181 (17)	0.2350 (5)	0.50843 (18)	0.0573 (7)	
H8	0.6262	0.2267	0.5122	0.069*	
C9	0.74793 (17)	0.2271 (5)	0.58380 (17)	0.0553 (7)	
H9	0.7362	0.2155	0.6375	0.066*	
C10	0.83122 (15)	0.2364 (4)	0.57930 (15)	0.0439 (6)	
C11	0.84728 (16)	0.2591 (4)	0.49868 (16)	0.0484 (6)	
H11	0.9028	0.2672	0.4946	0.058*	
C12	0.78090 (15)	0.2698 (4)	0.42436 (16)	0.0493 (6)	
C13	0.97201 (16)	0.2199 (4)	0.66068 (16)	0.0497 (6)	
H13	0.9866	0.2123	0.6083	0.060*	
C14	1.03864 (15)	0.2203 (4)	0.74123 (16)	0.0468 (6)	
C15	1.12379 (17)	0.2157 (5)	0.74914 (18)	0.0576 (7)	
H15	1.1471	0.2089	0.7020	0.069*	
C16	1.17241 (17)	0.2224 (4)	0.83561 (18)	0.0553 (7)	
H16	1.2313	0.2217	0.8532	0.066*	
C17	1.12169 (16)	0.2299 (4)	0.88979 (17)	0.0471 (6)	
N1	1.14919 (17)	0.2405 (4)	0.98236 (16)	0.0612 (6)	
N2	0.89429 (13)	0.2296 (3)	0.65958 (13)	0.0461 (5)	
O1	1.0953 (2)	0.2498 (4)	1.02221 (16)	0.0970 (9)	
O2	1.22480 (16)	0.2380 (4)	1.01700 (15)	0.0842 (8)	
O3	0.63071 (11)	0.2755 (3)	0.35573 (12)	0.0565 (5)	
S1	1.01578 (4)	0.22986 (10)	0.83989 (4)	0.0476 (2)	
Cl1	0.56369 (6)	−0.32087 (18)	0.08067 (6)	0.0923 (3)	
Cl2	0.80216 (5)	0.30835 (18)	0.32540 (4)	0.0828 (3)	

### Atomic displacement parameters (Å^2^)

**Table:**

	*U*^11^	*U*^22^	*U*^33^	*U*^12^	*U*^13^	*U*^23^
C1	0.0445 (14)	0.081 (2)	0.0543 (16)	−0.0028 (14)	0.0062 (12)	−0.0082 (16)
C2	0.0536 (16)	0.071 (2)	0.0631 (18)	0.0057 (14)	0.0019 (13)	−0.0003 (16)
C3	0.0503 (15)	0.072 (2)	0.0481 (15)	0.0038 (13)	−0.0069 (12)	0.0053 (14)
C4	0.0382 (12)	0.0660 (18)	0.0418 (13)	−0.0006 (12)	0.0046 (10)	0.0002 (13)
C5	0.0464 (14)	0.072 (2)	0.0451 (14)	0.0038 (13)	0.0017 (11)	0.0075 (14)
C6	0.0513 (15)	0.084 (2)	0.0438 (14)	0.0023 (14)	0.0001 (11)	0.0061 (15)
C7	0.0418 (12)	0.0553 (17)	0.0380 (12)	0.0032 (11)	0.0009 (10)	0.0002 (11)
C8	0.0408 (13)	0.082 (2)	0.0475 (15)	−0.0002 (12)	0.0088 (11)	−0.0007 (14)
C9	0.0497 (14)	0.079 (2)	0.0362 (13)	−0.0028 (13)	0.0088 (11)	0.0001 (13)
C10	0.0464 (13)	0.0454 (15)	0.0352 (12)	−0.0013 (10)	0.0025 (10)	−0.0007 (10)
C11	0.0405 (12)	0.0636 (18)	0.0378 (13)	−0.0019 (11)	0.0045 (10)	−0.0012 (12)
C12	0.0454 (13)	0.0637 (18)	0.0360 (12)	0.0001 (11)	0.0057 (10)	0.0005 (12)
C13	0.0521 (15)	0.0562 (16)	0.0359 (12)	−0.0013 (11)	0.0029 (11)	−0.0003 (11)
C14	0.0468 (13)	0.0489 (15)	0.0397 (12)	−0.0028 (11)	0.0027 (10)	0.0019 (11)
C15	0.0486 (14)	0.077 (2)	0.0458 (14)	−0.0048 (13)	0.0092 (11)	0.0037 (14)
C16	0.0419 (13)	0.0652 (19)	0.0530 (15)	−0.0026 (12)	0.0020 (11)	0.0035 (13)
C17	0.0466 (13)	0.0444 (15)	0.0427 (13)	−0.0035 (10)	−0.0014 (11)	0.0010 (11)
N1	0.0716 (16)	0.0571 (16)	0.0431 (12)	−0.0040 (12)	−0.0055 (12)	−0.0017 (11)
N2	0.0469 (11)	0.0520 (13)	0.0326 (10)	−0.0034 (9)	−0.0013 (8)	−0.0006 (9)
O1	0.106 (2)	0.138 (3)	0.0482 (12)	0.0076 (16)	0.0216 (13)	−0.0073 (14)
O2	0.0833 (16)	0.0886 (18)	0.0569 (13)	−0.0110 (12)	−0.0239 (11)	−0.0014 (11)
O3	0.0443 (9)	0.0723 (14)	0.0440 (10)	0.0095 (8)	−0.0039 (7)	−0.0035 (9)
S1	0.0430 (3)	0.0539 (4)	0.0422 (3)	−0.0016 (3)	0.0049 (2)	−0.0008 (3)
Cl1	0.0864 (6)	0.1102 (8)	0.0710 (6)	0.0071 (5)	0.0042 (4)	−0.0307 (5)
Cl2	0.0535 (4)	0.1552 (9)	0.0373 (4)	−0.0077 (4)	0.0079 (3)	0.0114 (4)

### Geometric parameters (Å, °)

**Table:**

C1—C6	1.378 (5)	C10—C11	1.388 (4)
C1—C2	1.378 (4)	C10—N2	1.419 (3)
C1—Cl1	1.736 (3)	C11—C12	1.383 (3)
C2—C3	1.391 (4)	C11—H11	0.9300
C2—H2	0.9300	C12—Cl2	1.725 (3)
C3—C4	1.381 (4)	C13—N2	1.269 (3)
C3—H3	0.9300	C13—C14	1.450 (3)
C4—O3	1.375 (3)	C13—H13	0.9300
C4—C5	1.376 (4)	C14—C15	1.366 (4)
C5—C6	1.390 (4)	C14—S1	1.713 (3)
C5—H5	0.9300	C15—C16	1.400 (4)
C6—H6	0.9300	C15—H15	0.9300
C7—C8	1.360 (4)	C16—C17	1.348 (4)
C7—O3	1.387 (3)	C16—H16	0.9300
C7—C12	1.390 (4)	C17—N1	1.428 (3)
C8—C9	1.391 (4)	C17—S1	1.707 (2)
C8—H8	0.9300	N1—O2	1.215 (3)
C9—C10	1.386 (4)	N1—O1	1.218 (4)
C9—H9	0.9300		
			
C6—C1—C2	121.2 (3)	C11—C10—N2	124.8 (2)
C6—C1—Cl1	119.4 (2)	C12—C11—C10	120.2 (2)
C2—C1—Cl1	119.3 (3)	C12—C11—H11	119.9
C1—C2—C3	118.5 (3)	C10—C11—H11	119.9
C1—C2—H2	120.7	C11—C12—C7	120.5 (2)
C3—C2—H2	120.7	C11—C12—Cl2	119.4 (2)
C4—C3—C2	120.3 (3)	C7—C12—Cl2	120.10 (19)
C4—C3—H3	119.9	N2—C13—C14	121.9 (3)
C2—C3—H3	119.9	N2—C13—H13	119.0
O3—C4—C5	116.1 (3)	C14—C13—H13	119.0
O3—C4—C3	123.0 (2)	C15—C14—C13	126.3 (3)
C5—C4—C3	121.0 (3)	C15—C14—S1	112.38 (19)
C4—C5—C6	118.9 (3)	C13—C14—S1	121.3 (2)
C4—C5—H5	120.6	C14—C15—C16	113.0 (3)
C6—C5—H5	120.6	C14—C15—H15	123.5
C1—C6—C5	120.1 (3)	C16—C15—H15	123.5
C1—C6—H6	119.9	C17—C16—C15	110.3 (2)
C5—C6—H6	119.9	C17—C16—H16	124.8
C8—C7—O3	119.7 (2)	C15—C16—H16	124.8
C8—C7—C12	119.3 (2)	C16—C17—N1	125.9 (2)
O3—C7—C12	120.7 (2)	C16—C17—S1	115.0 (2)
C7—C8—C9	120.7 (2)	N1—C17—S1	119.1 (2)
C7—C8—H8	119.6	O2—N1—O1	123.7 (3)
C9—C8—H8	119.6	O2—N1—C17	118.4 (3)
C10—C9—C8	120.4 (2)	O1—N1—C17	118.0 (3)
C10—C9—H9	119.8	C13—N2—C10	120.2 (2)
C8—C9—H9	119.8	C4—O3—C7	118.9 (2)
C9—C10—C11	118.9 (2)	C17—S1—C14	89.30 (12)
C9—C10—N2	116.3 (2)		
			
C6—C1—C2—C3	−1.6 (5)	N2—C13—C14—C15	−178.1 (3)
Cl1—C1—C2—C3	178.2 (2)	N2—C13—C14—S1	1.1 (4)
C1—C2—C3—C4	−0.1 (5)	C13—C14—C15—C16	178.5 (3)
C2—C3—C4—O3	−179.9 (3)	S1—C14—C15—C16	−0.7 (3)
C2—C3—C4—C5	1.8 (4)	C14—C15—C16—C17	0.6 (4)
O3—C4—C5—C6	179.9 (2)	C15—C16—C17—N1	−179.2 (3)
C3—C4—C5—C6	−1.6 (4)	C15—C16—C17—S1	−0.1 (3)
C2—C1—C6—C5	1.8 (5)	C16—C17—N1—O2	−1.9 (4)
Cl1—C1—C6—C5	−178.1 (2)	S1—C17—N1—O2	179.1 (2)
C4—C5—C6—C1	−0.1 (4)	C16—C17—N1—O1	178.8 (3)
O3—C7—C8—C9	−175.8 (3)	S1—C17—N1—O1	−0.2 (4)
C12—C7—C8—C9	−0.8 (4)	C14—C13—N2—C10	177.6 (2)
C7—C8—C9—C10	−0.9 (5)	C9—C10—N2—C13	173.8 (2)
C8—C9—C10—C11	1.7 (4)	C11—C10—N2—C13	−8.8 (4)
C8—C9—C10—N2	179.3 (3)	C5—C4—O3—C7	−163.4 (2)
C9—C10—C11—C12	−0.7 (4)	C3—C4—O3—C7	18.2 (4)
N2—C10—C11—C12	−178.1 (3)	C8—C7—O3—C4	−114.7 (3)
C10—C11—C12—C7	−1.0 (4)	C12—C7—O3—C4	70.5 (3)
C10—C11—C12—Cl2	177.3 (2)	C16—C17—S1—C14	−0.2 (2)
C8—C7—C12—C11	1.8 (4)	N1—C17—S1—C14	178.9 (2)
O3—C7—C12—C11	176.7 (2)	C15—C14—S1—C17	0.5 (2)
C8—C7—C12—Cl2	−176.5 (2)	C13—C14—S1—C17	−178.8 (2)
O3—C7—C12—Cl2	−1.6 (4)		

### Hydrogen-bond geometry (Å, °)

**Table:**

*D*—H···*A*	*D*—H	H···*A*	*D*···*A*	*D*—H···*A*
C3—H3···O2^i^	0.93	2.38	3.278 (3)	162.
C13—H13···O1^ii^	0.93	2.53	3.369 (4)	150.

Symmetry codes: (i) −*x*+2, *y*−1/2, −*z*+3/2; (ii) *x*, −*y*+1/2, *z*−1/2.
